# Stability of AI-Enabled Diagnosis of Parkinson’s Disease: A Study Targeting Substantia Nigra in Quantitative Susceptibility Mapping Imaging

**DOI:** 10.3389/fnins.2021.760975

**Published:** 2021-11-23

**Authors:** Bin Xiao, Naying He, Qian Wang, Feng Shi, Zenghui Cheng, Ewart Mark Haacke, Fuhua Yan, Dinggang Shen

**Affiliations:** ^1^School of Biomedical Engineering, Shanghai Jiao Tong University, Shanghai, China; ^2^Shanghai United Imaging Intelligence Co., Ltd., Shanghai, China; ^3^Department of Radiology, Ruijin Hospital, School of Medicine, Shanghai Jiao Tong University, Shanghai, China; ^4^School of Biomedical Engineering, ShanghaiTech University, Shanghai, China; ^5^Department of Radiology, Wayne State University, Detroit, MI, United States

**Keywords:** Parkinson’s disease, computer-assisted diagnosis, deep learning, stability, quantitative susceptibility mapping, radiomics

## Abstract

**Purpose:** Parkinson’s disease (PD) diagnosis algorithms based on quantitative susceptibility mapping (QSM) and image algorithms rely on substantia nigra (SN) labeling. However, the difference between SN labels from different experts (or segmentation algorithms) will have a negative impact on downstream diagnostic tasks, such as the decrease of the accuracy of the algorithm or different diagnostic results for the same sample. In this article, we quantify the accuracy of the algorithm on different label sets and then improve the convolutional neural network (CNN) model to obtain a high-precision and highly robust diagnosis algorithm.

**Methods:** The logistic regression model and CNN model were first compared for classification between PD patients and healthy controls (HC), given different sets of SN labeling. Then, based on the CNN model with better performance, we further proposed a novel “gated pooling” operation and integrated it with deep learning to attain a joint framework for image segmentation and classification.

**Results:** The experimental results show that, with different sets of SN labeling that mimic different experts, the CNN model can maintain a stable classification accuracy at around 86.4%, while the conventional logistic regression model yields a large fluctuation ranging from 78.9 to 67.9%. Furthermore, the “gated pooling” operation, after being integrated for joint image segmentation and classification, can improve the diagnosis accuracy to 86.9% consistently, which is statistically better than the baseline.

**Conclusion:** The CNN model, compared with the conventional logistic regression model using radiomics features, has better stability in PD diagnosis. Furthermore, the joint end-to-end CNN model is shown to be suitable for PD diagnosis from the perspectives of accuracy, stability, and convenience in actual use.

## Introduction

Parkinson’s disease (PD) is a significant neurodegenerative disease (Damier et al., 1999). The main symptoms of PD include static tremor, bradykinesia, and myotonia. As the clinical manifestations of PD are highly diverse among individual patients, the diagnosis heavily depends on domain knowledge and experience of the clinicians (Rodriguez-Oroz et al., 2009). At present, the diagnosis process is typically based on clinical assessment, which can be very time-consuming. From the first clinic visit to finally reaching a diagnosis, it may take months to sometimes even several years. Since the delay of diagnosis can be detrimental to properly treating the patients, it is crucial to shorten the time to derive a correct PD diagnosis.

Among many medical imaging modalities, magnetic resonance imaging (MRI) has excellent soft-tissue contrast and can be used to reveal the differences between PD patients and healthy controls (HCs) based on the presence of abnormal image cues (Tessa et al., 2014; Guan et al., 2017; Zeng et al., 2017; Shu et al., 2020)—for instance, excessive iron deposition in the substantia nigra (SN) regions of PD patients has become a strong candidate for PD biomarkers (Haller et al., 2013). Particularly, quantitative susceptibility mapping (QSM), as a newly emerging tool to measure iron content in the basal ganglia (Shahmaei et al., 2019), can visualize the high amount of iron in deep gray matter clearly (He et al., 2017).

The intensity values in QSM images can be used as quantitative descriptions for PD research (Ghassaban et al., 2019; He et al., 2021). Intuitively, the more iron deposition there is, the larger the intensity value in the QSM image (Langkammer et al., 2012). Furthermore, for early PD patients, the midbrain black iron deposit has been significantly increased, rendering a high correlation with the severity of the motion symptoms of the patients (He et al., 2015). Therefore, it is necessary and feasible to investigate automatic diagnostics based on QSM to shorten and quantify the diagnosis process.

Technically, there are two subtasks that are coupled in addressing the QSM-based computer-assisted diagnosis of PD: (1) SN labeling, for locating the region of interest, and (2) feature extraction and classification, as detailed below:

(1) For SN labeling, one may employ either manual labeling of SN or automatic segmentation (Dong et al., 2016; Zhang et al., 2016). Manual labeling is often perceived as gold standard in annotating medical images and widely adopted in many studies, but the process is very time-consuming. In order to reduce the time cost and importantly get rid of the dependence on objective manual labeling in practical application, automatic segmentation of SN has provided an attractive alternative. The SN segmentation based on iterative optimization (Visser et al., 2016a,b; Guo et al., 2018) can achieve 75–79% in Dice score. Due to the high contrast provided by QSM, Garzon et al. (2018) proposed an SN segmentation method based on multi-atlas registration. By registering and fusing multiple atlases, an 81% Dice score is reached for SN segmentation.
(2) For feature extraction and classification, enabled by sophisticated machine learning methods, one may develop automatic diagnosis methods based on QSM imaging data and SN labeling. In addition to first-order features such as the mean value of image intensities, researchers extract 105 radiomics features to characterize iron deposition changes within the SN (Cheng et al., 2019). After feature selection, the support vector machine model is employed to complete the classification between idiopathic PD and HC and reaches an accuracy of 88%. In our previous work, we apply a convolutional neural network (CNN) to the entire SN region for feature extraction and PD/HC classification (Xiao et al., 2019). We further combine CNN features and conventional radiomics features into a hybrid framework and obtain 90% accuracy in PD diagnosis. All these results demonstrate that the machine learning algorithm can solve this clinical usage effectively.

**FIGURE 1:**
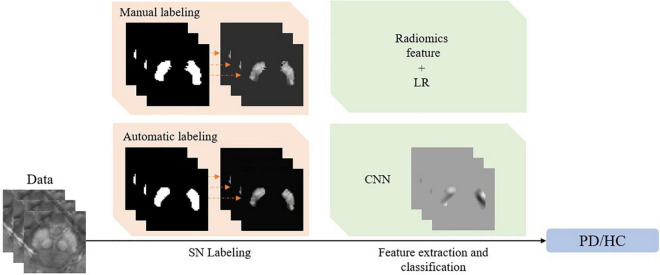
The framework of the quantitative susceptibility mapping-based Parkinson’s disease diagnosis model.

Based on the achievement mentioned above, Figure 1 summarizes the current typical framework for QSM-based PD diagnosis, consisting of two consecutive steps. In the first step of SN labeling, one may adopt conventional manual labeling or automatic labeling algorithms. In the second step of feature extraction and classification, one may extract radiomics features given the labeled SN regions and then use the classical logistic regression (LR) model to complete the diagnosis. Alternatively, we can use CNN to complete the whole diagnosis task automatically.

However, a problem that is often neglected in the abovementioned methods is that SN labeling usually comes from different sources, and the slight difference in SN labeling could considerably affect classification accuracy and consistency. Hence, it is essential to quantify and improve the robustness of the abovementioned diagnostic model regarding labeling variation. The model is expected to meet the following two requirements: (1) A model trained well on manual labeling should not have obvious performance degradation on automatic labeling, as automatic labeling is convenient to use after deploying the trained model to clinical practice; and (2) the trained model should be robust and make same judgment on the same patient, even though two different sources of labeling (e.g., from two different doctors) are considered.

As such, this study aims to prospectively investigate the stability of the abovementioned PD diagnosis model, especially with respect to SN labeling. Specifically, we divide our work into the following three parts:

(1) We first compare two different classification schemes, i.e., LR and CNN, when different sets of SN labeling are provided. The goal here is to verify whether CNN, a recently popular approach, can deliver better stability than the classical LR model in PD/HC classification, given SN contours of various sources.
(2) We further propose a novel “gated pooling” operation and integrate it with the CNN model. We show that the CNN model can then effectively improve the PD/HC classification performance, in which gated pooling can further mitigate the instability caused by SN labeling.
(3) Finally, we combine the two steps of labeling and classification into a unified CNN model, with the help of gated pooling. We demonstrate that the end-to-end deep learning achieves not only accurate but also stable PD diagnosis, regardless of manual or automatic labeling of the SNs.

Based on these three parts, the final aim is to quantify the stability of the current diagnosis algorithm and provide a fully automatic PD/HC diagnosis solution that is highly accurate, robust, and repeatable.

## Materials and Methods

We first prepare the data and set up the models to be analyzed in sections “Data Collection” and “Setup of Labeling and Classification Models,” respectively. Then, in section “Classification Stability Due to Labeling” and “Classification Intrasubject Instability Due to Labeling,” the stability analysis with respect to SN labeling is detailed. In section “Joint Learning for Labeling and Classification,” we further combine the two steps of labeling and classification into a unified deep learning framework.

### Data Collection

This study was approved by the local ethics committee in Ruijin Hospital, Shanghai Jiao Tong University School of Medicine. All participants provided written informed consent. The recruitment and MRI scanning of the participant are the same as shown in a prior study (He et al., 2020). In this study, in total, 87 right-handed PD participants (age: 60.9 ± 8.1 years; man/woman: 41/46) from local movement disorder outpatient clinics were recruited. All PD participants were diagnosed according to the United Kingdom Parkinson’s Disease Society Brain Bank criteria (Hughes et al., 1992). Demographic information, including sex, age, and education, was collected for each participant. Disease severity was evaluated using Hoehn and Yahr staging, and motor disability was assessed using the motor portion of the Unified Parkinson’s Disease Rating Scale—III in the ON medication state. The inclusion criteria for the PD group were a diagnosis of idiopathic PD. The exclusion criteria were as follows: (1) secondary parkinsonism which was caused by the use of drugs (e.g., antipsychotics, antiemetics, and drugs that deplete dopamine) and atypical parkinsonian symptoms (such as progressive supranuclear palsy, multiple system atrophy, dementia with Lewy bodies, and corticobasal syndrome); (2) Mini-Mental State Exam (MMSE) score lower than 24; (3) a history of cerebrovascular disease, seizures, brain surgery, brain tumor, moderate-to-severe head trauma, or hydrocephalus; or (4) treatment with antipsychotic drugs or with any other drug possibly affecting the clinical evaluation. For HCs, 53 sex- and age-matched right-handed participants (age: 62.9 ± 7.1 years; man/woman: 24/29) were recruited from the local community. The inclusion criteria for the control group were as follows: (1) normal movement function and neurological status, (2) absence of neurological or psychiatric disease, and (3) a MMSE score equal or greater than 24. The demographic and clinical characteristics are shown in Supplementary Table 1.

All participants were imaged with a 3T scanner (Signa HDxt; GE Healthcare) equipped with an eight-channel receive-only head coil. A 3D multi-echo gradient echo (GRE) sequence was used to acquire images suitable for measurement of R2* with the following parameters: repetition time (TR) = 59.3 ms, number of echoes = 16, first echo time = 2.7 ms, echo spacing = 2.9 ms, flip angle (FA) = 12°, field of view = 220 × 220 mm^2^, resolution = 0.86 × 0.86 × 1.0 mm^3^, sensitivity encoding acceleration factor = 2, and total acquisition time = 10 min, 42 s. Whole-brain anatomical images were acquired with a T1-weighted fast-spoiled GRE sequence for common space registration. The imaging parameters for this sequence were as follows: TR = 5.5 ms, TE = 1.7 ms, inversion time = 450 ms, resolution = 1 × 1 × 1 mm^3^, and FA = 12°.

The QSM image reconstruction was performed according to a previous study (Li et al., 2011). Briefly, all phase images were averaged and filtered with SHARP, and the susceptibility maps were derived from the frequency map iLSQR (the regularization threshold for Laplace filtering was set at 0.04) (Li et al., 2011). The unit of susceptibility was given in parts per billion. As a summary, the data and subjects used here were the same with our previous work (Xiao et al., 2019).

### Setup of Labeling and Classification Models

There are two major ways for SN labeling and PD/HC classification, respectively, in this study. We set up the corresponding labeling and classification (including feature extraction) implementations in advance.

First, the labeling step can either accommodate manual labeling or be adopted by the cutting-edge learning-based segmentation tools. For manual labeling particularly, the contour of the SN of our data was drawn manually by a neuroradiologist with 10 years of neuroimaging experience. The expert labeling is regarded as ground truth. Then, for automatic labeling, we adopt and improve V-net to achieve a state-of-the-art performance (Dong et al., 2016; Visser et al., 2016a,b; Garzon et al., 2018; Guo et al., 2018). The architecture of V-net (Milletari et al., 2016) has been widely used in various medical image segmentation tasks. In our implementation, we delete all pooling operations in V-net, which reduces image spatial resolution and may hurt the contouring precision of SN. Meanwhile, we have replaced all residual blocks (He et al., 2016) with densely connected blocks (Huang et al., 2017), which further promotes visual feature fusion at various scales in the network (Jegou et al., 2017).

**FIGURE 2:**
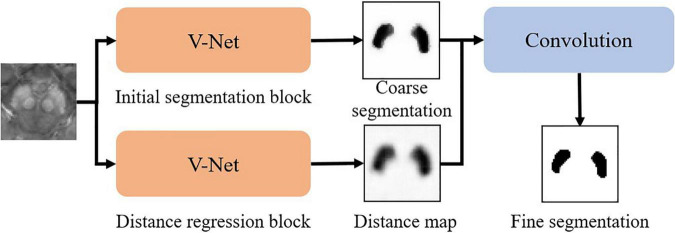
The architecture of SN^2^ used for substantia nigra labeling.

The whole pipeline of our SN segmentation network (namely SN2) follows a robust coarse-to-fine manner as in Figure 2. The coarse segmentation includes two parallel modules, i.e., (1) initial segmentation block and (2) distance regression block, both of which follow the same V-net architecture. The initial segmentation block outputs the probability map of SN. The distance regression block estimates the distance from each point in the image to the nearest boundary of SN. If a point is inside SN, the sign of the distance is then negative. If the point is outside SN, the distance is positive. By using both blocks, we can suppress possible false-positive errors in the segmentation results. The outputs of the initial segmentation block and the distance regression block are concatenated in the channel dimension for further convolution, followed by softmax to derive the fine segmentation.

Our experiments show that our SN2 can achieve the Dice coefficient of 87.2%, compared to 83% of the original V-Net. The Dice coefficient, which is a typical indicator for segmentation quality, measures how close the outputs of SN2 are compared to the ground-truth labeling of the expert radiologist. To our knowledge, the Dice coefficient by SN2 is superior to most existing automatic labeling (Garzon et al., 2018). For convenience, we denote the manual labeling as *L*_*1.000*_ and the outputs of SN2 as *L*_*0.872*_, with the subscripts indicating the corresponding Dice coefficients with respect to the ground-truth labeling.

**FIGURE 3:**
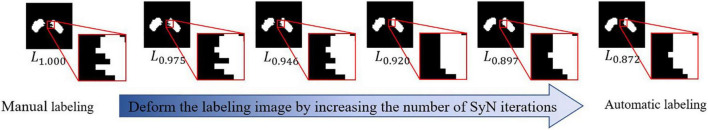
Examples of the substantia nigra labels in different sets.

In order to investigate the impact of labeling upon feature extraction and classification, we need to generate a series of SN labeling with a different precision, which can help us track the effects of SN labeling on the performance of PD/HC diagnosis. Using a popular registration algorithm SyN (Avants et al., 2008), we can obtain a deformation field that can align *L*_*1.000*_ and *L*_*0.872*_. By changing the magnitude of this deformation field (i.e., by changing the iterative callbacks in SyN) and apply them to warp*L*_1.000_, we can generate a set of SN labeling with various precisions. Specifically, the resulting SN labeling sets are *L*_*0.975*_, *L*_*0.946*_, *L*_*0.920*_, and *L*_*0.897*_, where different subscripts show the corresponding Dice coefficient relative to*L*_1.000_. Since the deformation field is smooth, the SN sets can be perceived as a sequence of gradual deviation from manual labeling to automatic labeling. We visualize a typical example of the abovementioned process in Figure 3.

**FIGURE 4:**
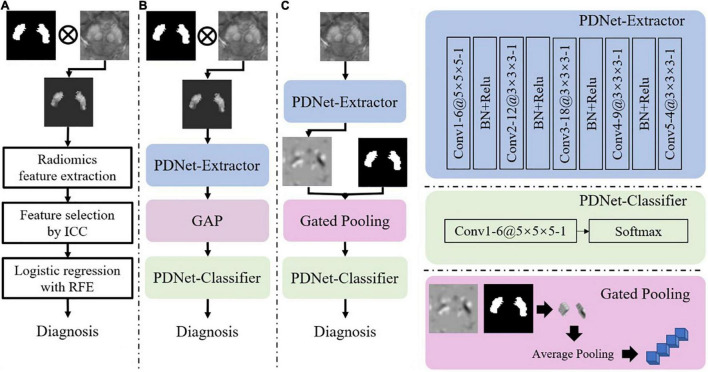
Illustrations of implementation schemes for feature extraction and classification for PD/HC diagnosis. **(A)** Radiomics features + LR: features are extracted from QSM images with SN labels (ICC: intraclass correlation coefficient, RFE: recursive feature elimination). **(B)** PDNet: a deep learning network consisting of feature extractor and classifier, which are further detailed in the right panel of the figure. **(C)** PDNet + Gated Pooling: the Gated Pooling operation is integrated with PDNet, which is also detailed in the right-bottom of the figure, for better classification performance. “Conv”, “BN” and “GAP” stand for convolution, batch normalization, and global average pooling, respectively, in the figure. The number of channels (e.g., 6), kernel size (e.g., 5 × 5 × 5), and stride (e.g., 1) in each convolution layer of PDNet are shown as “6@5 × 5 × 5 -1”.

Second, we consider two options for feature extraction and classification, i.e., radiomics features + LR and CNN-based PDNet. The two options, which are illustrated in Figures 4A,B, have both shown effectiveness for PD/HC classification. As in Figure 4A, the LR model relies on radiomics features. Given two sets of SN labeling (e.g., *L*_*1.000*_ and L_*_∈{*L*_1.000_,*L*_0.975_,*L*_0.946_,*L*_0.920_,*L*_0.897_,*L*_0.872_}), we first mask out the SN region from the original QSM image and then extract radiomics features using Pyradiomics (van Griethuysen et al., 2017). In total, 2,210 first-order shape and texture radiomics features in seven categories are extracted, including the following: first-order, *n* = 432; shape feature, *n* = 26; gray-level co-occurrence matrix, *n* = 24; gray-level dependence matrix, *n* = 528; gray-level run-length matrix, *n* = 384; gray-level size-zone matrix, *n* = 384; and neighborhood gray-tone difference matrix, *n* = 120. Detailed descriptions of these radiomics features are available at https://pyradiomics.readthedocs.io/en/latest/index.html. To obtain the radiomics feature with high stability between *L*_*1.000*_ and L_*_, intraclass correlation coefficients (ICC) (2,1) estimates are calculated on these feature values based on the assumption of single rater, absolute agreement, and two-way random effects (Koo and Li, 2017). A subset of these radiomics features with ICC > 0.8 is preserved. Then, we use the LR model combined with the recursive feature elimination strategy to complete the final feature selection and model training on the features extracted from *L*_1.000_.

For the CNN-based PDNet model (c.f. Figure 4B), we can divide it into PDNet-Extractor and PDNet-Classifier, respectively. PDNet-Extractor encodes the input image (masked by the SN labeling first) to a multi-channel feature maps, which avoids using the human-engineered radiomics features as in the previous LR model. Note that PDNet-Extractor considers image cues in the SN region only since the region contains critical information to PD diagnosis. Given the feature maps from PDNet-Extractor, PDNet-Classifier can complete the classification task and derive PD diagnosis after proper pooling operation (e.g., global average pooling, or GAP, in Figure 4B). The detailed network structure of PDNet is shown in Figure 4.

### Classification Stability Due to Labeling

It is hypothesized that the stability of PD/HC classification, as a second step in the pipeline of Figure 1, highly depends on the quality of the SN labeling in the first step. To quantitatively investigate the impact of labeling upon feature extraction and classification, we need to investigate the accuracy of the classification model on different SN labeling. First of all, we prepare the well-trained diagnosis model (Figures 4A,B) on *L*_*1.000*_ as described in section “Setup of Labeling and Classification Models.” After that, by applying L_*_∈{*L*_1.000_,*L*_0.975_,*L*_0.946_,*L*_0.920_,*L*_0.897_,*L*_0.872_} to the classification models, we can then track the impact of SN labeling on the performance of PD/HC diagnosis. The performance comparisons between the models in Figures 4A,B are summarized in section “Population-Level Classification Stability.”

### Classification Intrasubject Instability Due to Labeling

While our later experimental results suggest that PDNet delivers higher PD/HC classification accuracy than the conventional LR model in the overall population level, the deep learning network still suffers from intrasubject instability. Given the gradually changing SN labeling, the same subjects can be correctly classified sometimes yet fail at other times. The inconsistent classification becomes a significant challenge when applying a diagnosis model to practice since it is unknown what kind of SN labeling is needed per patient.

The abovementioned inconsistency may originate from the way the SN labeling is used by PDNet. Following the approach in Xiao et al. (2019), we can set the region out of SN to zero, which makes the edges of SN have a sharp contrast in the input to PDNet. The sharp contrast pushes the convolution to produce active output, which dramatically affects the decision-making process of the CNN model. Therefore, the edge appearance of SNs in different labeling sets contribute highly to the inconsistency of classification.

To mitigate this issue, we propose the gated pooling operation and improve the PDNet as in Figure 4C. We input the original QSM image nearby the SN region to the PDNet-Extractor. On the feature maps from PDNet-Extractor, we collect the high-order features within the SN labeling and perform average pooling to derive a feature vector. The abovementioned process, which is termed as gated pooling, replaces GAP compared to Figure 4B. After gated pooling, the feature vector is sent to the PDNet-Classifier for classification. With Gated Pooling, the SN edge impact can be effectively alleviated.

### Joint Learning for Labeling and Classification

Since Gated Pooling can bridge labeling and classification, we further implement a unified CNN model for joint learning of the labeling and classification tasks in an end-to-end fashion.

**FIGURE 5:**
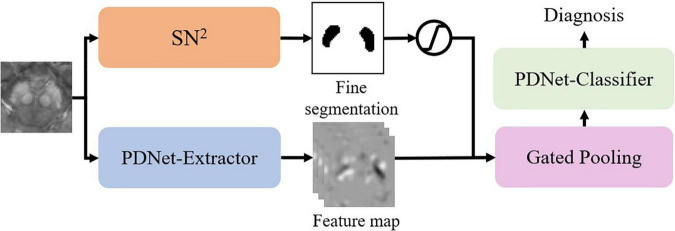
Unified deep learning framework for the automatic diagnosis of Parkinson’s disease.

As in Figure 5, we integrate SN2 into the classification model by gated pooling. Note that the fine segmentation is represented by the probability map. We fuse the probability map to the feature maps of the PDNet-Extractor in the following way:


feature⁢vec⁢tor≜mean⁢(δ⁢(p)×feature⁢map),

$$\text{where}\,\delta \,\left(p\right)=\left\{ \begin{array}{cc} 1 & p>0.6,\\ 5\,p-2 & 0.6\geq p>0.4,\\ 0 & p\leq 0.4. \end{array}\right.$$

Here p is the probability of the voxel belonging to SN. We can view this operation as a different version of gated pooling. After that, the PDNet-Classifier maps the feature vector to a diagnostic result. We can train the abovementioned network in an end-to-end manner.

## Experimental Validation

Following the same experimental setting as that of Xiao et al. (2019), we have performed a sevenfold nested cross-validation. For the 140 subjects collected, we randomly select 100 subjects as the training set, 20 subjects as the validation set, and the rest of the 20 subjects as the testing set in each fold. The validation set is used for hyper-parameter tuning only, and the testing set is used for performance evaluation. The nested cross-validation is permutated 50 times, and the measures reported below are collected from all 50 permutations.

### Population-Level Classification Stability

To verify that the CNN-based PDNet can deliver better stability than LR in PD/HC classification, we train the radiomics features + LR model (Figure 4A) and PDNet model (Figure 4B) on the labels of *L*_*1.000*_. Then, we test with different sets of labels generated in section “Setup of Labeling and Classification Models.”

**FIGURE 6:**
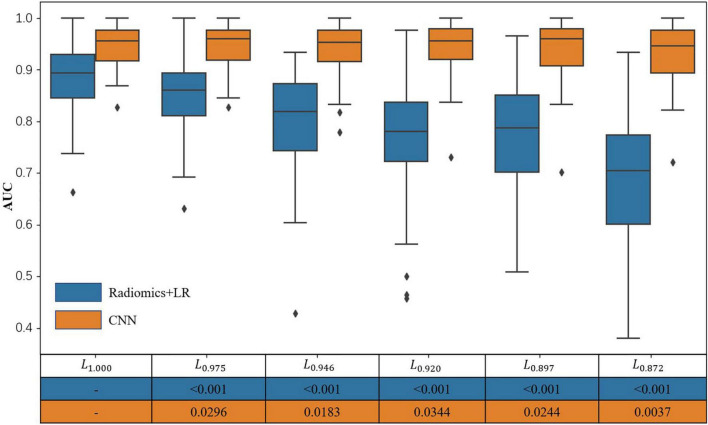
Classification areas-under-curve of logistic regression and PDNet.

It can be seen from Figure 6 and Table 1 that, as the SN labeling deviates from *L*_*1.000*_ with decreasing Dice scores, the classification accuracy of the LR model based on radiomics features has a clear downward trend. This implies that LR model based on the radiomics feature is not robust to the differences between different SN labeling. The CNN-based classification model, on the other hand, yields a relatively good stability across different sets of SN labeling. The produced classification areas-under-curve (AUCs), for example, are fluctuating slightly, given different SN labeling with different precision. All these phenomena enlighten us that, in the practical application of the classification model based on radiomics features, we need to further consider the impact caused by different labeling. On the contrary, the CNN-based model is less sensitive to the variation induced by the SN labeling, while its classification accuracy can be attained in a more stable way.

**TABLE 1:** Accuracy, area under the curve (AUC), balance accuracy (BAC), sensitivity, and specificity for LR and PDNet.

Dice	Accuracy (Mean ± Std)		AUC (Mean ± Std)		BAC (Mean ± Std)		Sensitivity (Mean ± Std)		Specificity (Mean ± Std)	
	LR	PDNet	LR	PDNet	LR	PDNet	LR	PDNet	LR	PDNet
1.000 (L_1.000_)	0.789 ± 0.081	0.864 ± 0.072	0.881 ± 0.066	0.947 ± 0.038	0.794 ± 0.081	0.865 ± 0.069	0.881 ± 0.077	0.926 ± 0.063	0.707 ± 0.127	0.803 ± 0.116
0.975 (L_0.975_)	0.764 ± 0.092	0.870 ± 0.059	0.850 ± 0.077	0.948 ± 0.040	0.763 ± 0.092	0.870 ± 0.057	0.833 ± 0.078	0.929 ± 0.064	0.692 ± 0.134	0.812 ± 0.100
0.946 (L_0.946_)	0.738 ± 0.094	0.853 ± 0.068	0.797 ± 0.103	0.945 ± 0.046	0.736 ± 0.101	0.856 ± 0.067	0.805 ± 0.082	0.926 ± 0.071	0.668 ± 0.150	0.787 ± 0.114
0.920 (L_0.920_)	0.721 ± 0.091	0.860 ± 0.072	0.766 ± 0.115	0.946 ± 0.050	0.716 ± 0.104	0.861 ± 0.068	0.784 ± 0.091	0.933 ± 0.064	0.648 ± 0.149	0.790 ± 0.115
0.897 (L_0.897_)	0.714 ± 0.101	0.860 ± 0.070	0.769 ± 0.109	0.941 ± 0.049	0.705 ± 0.108	0.861 ± 0.068	0.776 ± 0.088	0.923 ± 0.066	0.634 ± 0.144	0.800 ± 0.116
0.872 (L_0.872_)	0.679 ± 0.103	0.849 ± 0.083	0.695 ± 0.129	0.931 ± 0.052	0.665 ± 0.113	0.853 ± 0.077	0.736 ± 0.085	0.919 ± 0.067	0.594 ± 0.154	0.788 ± 0.130

*The models are trained with the labels in L_1.000_, yet they are tested with different sets of labels, corresponding to the gradually decreasing dice coefficients.*

### Intrasubject Classification Consistency

When using different testing labeling with a trained classification model, the same subject may get different diagnosis results, which is a manifestation of intrasubject instability. To quantify this instability, we define the classification consistency index (CCI). First, if a subject yields the same diagnosis result given two available SN labels, we count the subject as “1,” otherwise it is “0.” Then, we summarize all subjects in a testing set and compute the CCI measure by normalizing upon the size of the testing set. In this way, CCI tells the diagnosis consistency of a certain classifier when tested with multiple subjects, if the testing labels are generated in two different ways.

The upper triangle of Table 2 shows the CCIs of PDNet in Figure 4B. For any pair of labeling in{*L*_1.000_,*L*_0.975_,*L*_0.946_,*L*_0.920_,*L*_0.897_,*L*_0.872_}, PDNet suffers from intrasubject inconsistency to a certain extent. Taking the number of CCI(*L*_0.872_,*L*_1.000_)=0.926 in the upper right corner of Table 2 as an example, we trained 50 models as shown in Figure 4B through different data splits. Then, on the test set (20 test samples in each split, 50 * 20 = 1,000 samples for all 50 models), the SN area was extracted by the labeling with *L*_*1.000*_ accuracy (Dice = 1.000 in the Y-coordinate) and *L*_*0.872*_ accuracy (Dice = 0.827 in the X-coordinate), respectively, and then sent to the corresponding trained model. Only about 926 samples out of the 1,000 test samples shown have the same classification results under the two SN labels, which corresponds to 0.926 (926/1,000) in the upper-right corner of Table 2. The values in the upper-right section of Table 2 can be interpreted in the same way. It can be found that the model in Figure 4B will be affected by the difference between labeling. This obviously hinders the application of the algorithm in real scenarios.

**TABLE 2:** Classification consistency for two sets of labeling.

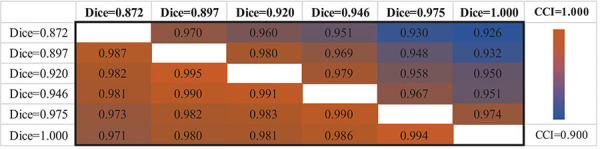

*For models with or without gated pooling, we calculate the logistic regression (CCIs) using any pair of label sets to extract substantia nigra (SN)—for example, theCCI(L_1.000_,L_0.872_) in the upper-right corner means the classification consistency index when we useL_1.000_ andL_0.872_ to extract SN. The CCIs in the upper triangle and lower triangle are corresponding to the models in Figures 4B,C, respectively. The closer the color is to orange, the closer the value is to 1 and the closer the color is to blue, the closer the value is to 0.9.*

Following the same experimental setup detailed above, we verify PDNet after integrating “gated pooling” (c.f. the model in Figure 4C). The results are then provided in the lower triangle of Table 2 accordingly. Corresponding to the CCI(*L*_0.872_,*L*_1.000_)=0.926 of Figure 4B, our proposed method in Figure 4C get CCI(*L*_1.000_,*L*_0.872_)=0.971 in the lower left corner of Table 2, which is higher than the number of 926 for the model in Figure 4B.

In summary, all CCIs from Figure 4C has increased in comparison to those in Figure 4B, indicating the contribution of “gated pooling” in boosting intrasubject classification consistency.

### Unified Framework for Automatic Diagnosis

Based on section “Joint Learning for Labeling and Classification,” we implement an end-to-end diagnosis framework (as in Figure 5) combining both labeling and classification tasks together. We aim to prove that an end-to-end system is better than independently calling segmentation and classification tasks. For comparison, we have trained multiple PDNets integrating gated pooling for PD/HC classification (as in Figure 4C). Their differences come from using different training settings and labels, with the detailed configurations shown in Table 3.

**TABLE 3:** Comparisons of different diagnosis networks.

SN labels used in training/in testing/network architecture	Accuracy (mean/Std/*p*-value)	AUC (mean/Std/*p*-value)	BAC (mean/Std/*p*-value)	Sensitivity (mean/Std/*p*-value)	Specificity (mean/Std/*p*-value)
L_1.000_/L_1.000_/Figure 4C	0.872/0.064/0.004	0.944/0.042/<0.001	0.877/0.062/0.019	0.923/0.079/0.002	0.831/0.110/0.175
L_1.000_/L_0.872_/Figure 4C	0.859/0.061/–	0.931/0.049/–	0.866/0.061/–	0.904/0.078/–	0.829/0.116/–
L_0.872_/L_0.872_/Figure 4C	0.827/0.075/0.004	0.921/0.052/0.053	0.832/0.073/0.002	0.890/0.089/0.087	0.775/0.119/0.003
L_1.000_/none/Figure 5	0.869/0.057/0.017	0.936/0.045/0.016	0.872/0.057/0.057	0.910/0.077/0.088	0.834/0.098/0.131

*We have trained four logistic regression models for comparative experiments. There are two options for extracting the substantia nigra: manual labeling (L_1.000_) or automatic labeling (L_0.872_). For training the network, we have two options: (1) training the segmentation network and classification network separately as in Figure 4C and then combining them together for testing and (2) end-to-end training as shown in Figure 5. As an example, the model in Figure 4C trained on the label set L_1.000_ and tested on L_0.872_ is denoted as “L_1.000_/L_0.872_/Figure 4C”. The p-value has been updated by the multiple-comparison correction.*

• Row 1: The PDNet adopts expert labeling in both training and testing—that is, if this network is applied to clinical practice for PD/HC diagnosis, an expert will have to label the SN regions for the entire training set in advance. Then, given a new patient for test, the (same) expert needs to label the SN region following the same protocol with the training data.
• Row 2: A more convenient way in practice is that the expert labels the SN regions for the training set, from which not only the classification task but also the segmentation task can be trained. Thus, in the testing stage, a new patient will not be manually labeled; the SN label will instead be generated automatically (i.e., at the quality level corresponding to *L*_*0.872*_).
• Row 3: We also replace the labels in training PDNet with the automatic labeling results. By comparing with row 1, we can further verify the impact when the SN labels are generated in an inconsistent way during the training and testing of PDNet.
• Row 4: The proposed unified architecture not only considers classification (as in the three previous rows) but also integrates segmentation. Thus, expert labeling is needed only in training. In testing, the patient will be labeled automatically inside the network, without need of external input.

Referring to PDNet in Figure 4C, the model trained and tested on both the expert labeling performs well (row 1 in Table 3). By using the automatic labeling to replace the expert labeling for test (row 2 in Table 3), the PDNet suffers from a slight degradation of the classification performance (i.e., the classification accuracy dropping from 0.872 ± 0.064 to 0.859 ± 0.061, *p* = 0.004, from matched-samples *t*-test after multiple-comparison correction). Here the results reported in Table 3 are collected from sevenfold nested cross-validation with 50 permutations. This performance drop is reasonable because the test labels are of different quality with the training labels as in row 2.

By using our proposed method in Figure 5, the accuracy increases from 0.859 ± 0.061 (row 2 in Table 3) to 0.869 ± 0.057 (row 4 in Table 3, *p* = 0.017 from matched-samples *t*-test after multiple-comparison correction). Note that our approach eliminates the performance drop, which can be attributed to the joint training of segmentation network and classification network. Meanwhile, no external labeling is required for the entire diagnostic process, which makes our method convenient to use if deployed in clinical practice.

A more meaningful comparison is between row 1 and row 4. Our proposed method can achieve the classification accuracy that is comparable to the PDNet with expert labeling in both training and testing (row 1 in Table 3). While no accuracy difference is detected statistically, our method does not need human participation in the whole diagnosis process, which eliminates the influence of subjective factors on the diagnosis results. This provides a feasible scheme for the application of the algorithm in real scenes.

In row 3, we replace the SN labeling in the training stage with the automatic labeling results. Compared to row 1, the classification performance further drops (i.e., 0.872 ± 0.064 vs. 0.827 ± 0.075 in accuracy, *p* = 0.004 from matched-samples *t*-test after multiple-comparison correction). The results imply that precise expert labeling is critical to train a well-functioning classification model.

## Discussion and Conclusion

The wider clinical use of QSM-based classification algorithm for PD/HC is contingent on understanding the robustness of the combination of SN labeling, image feature extraction, and classification algorithm. The relatively poor performance of “radiomics + LR” can be explained by two factors: (1) the high sensitivity of radiomics features on small target regions (Li et al., 2019) and (2) the low robustness of the LR model in handling different sources of region labeling. SN is a tiny basal nucleus, and the area around the edge of SN has sharp contrast in image cues. Influenced by this, the intensity-based radiomics features have significant fluctuations, even though the region labeling changes subtly. The LR model, which is modeled as a shallow combination of the radiomics features, obviously suffers from the instability of extracted features.

For the CNN model, the PDNet (Figure 4B) shows a high classification accuracy in section “Population-Level Classification Stability,” proving that the image features learned automatically through the network are better than the radiomics features. Simultaneously, PDNet (Figure 4C) integrated with “gated pooling” performs better in classification consistency. However, due to the lack of cooperation between SN labeling and PD diagnosis, the simple combination still brings some performance degradation. Therefore, it is necessary to build a unified end-to-end framework integrating segmentation and diagnosis for the final clinical application, as shown in Figure 5.

This study still has some limitations. The amount of data used in this article is limited, and we also did collect an independent test set. These may affect the generalization performance of our conclusions. For this, we will collect more data for further verification in the follow-up work and improve our CNN framework (Zhang et al., 2017).

Meanwhile, this study has only used a single-center dataset. As QSM imaging technology progresses continuously, there are several different protocols and reconstruction methods, which may affect the diagnosis algorithm. Practically, it is not possible to collect datasets for all settings and then use the collected data to tune the learning-based diagnosis model. The common features underlying the disease of PD are instead expected to be captured, while the diagnosis model can be trained more robustly through “domain adaptation”—that is, one may transfer the learned image features from existing datasets to a new set, such that the data from different domains or sources can be mixed together for larger data size and better modeling.

In general, this work shows that the CNN model, compared with the LR model based on radiomics features, has better stability in different SN labeling sources. Furthermore, the gated pooling operation provides the CNN model with higher prediction consistency without losing classification accuracy. Benefitting from this capability, our proposed unified framework for automatic diagnosis network in Figure 5 achieves a state-of-the-art performance in terms of accuracy, stability, and prediction consistency.

## Data Availability Statement

The raw data supporting the conclusions of this article will be made available by the authors, without undue reservation.

## Ethics Statement

The studies involving human participants were reviewed and approved by the Ethics Committee of Ruijin Hospital Affiliated to Shanghai Jiao Tong University School of Medicine. Written informed consent for participation was not required for this study in accordance with the national legislation and the institutional requirements.

## Author Contributions

BX and NH contributed to writing the original draft, review and editing, investigation, data curation, and formal analysis. QW, FS, ZC, EH, FY, and DS contributed to formal analysis, review and editing, conceptualization, supervision, funding acquisition, and resources. All authors contributed to the article and approved the submitted version.

## Conflict of Interest

FS and DS were employed by Shanghai United Imaging Intelligence Co., Ltd., Shanghai, China. The company has no role in designing, performing the surveillances, analyzing, and interpreting the data. The remaining authors declare that the research was conducted in the absence of any commercial or financial relationships that could be construed as a potential conflict of interest.

## Publisher’s Note

All claims expressed in this article are solely those of the authors and do not necessarily represent those of their affiliated organizations, or those of the publisher, the editors and the reviewers. Any product that may be evaluated in this article, or claim that may be made by its manufacturer, is not guaranteed or endorsed by the publisher.
