# Supplementary material for: Stability of AI-Enabled Diagnosis of Parkinson’s Disease: A Study Targeting Substantia Nigra in Quantitative Susceptibility Mapping Imaging
Source: Front Neurosci. 2021 Nov 23;15:760975. doi: 10.3389/fnins.2021.760975 (PMC8650720; doi:10.3389/fnins.2021.760975)
Supplement: Supplementary file 1 [file Table_1.docx]

*Supplementary Material for: Stability of AI-enabled diagnosis of Parkinson's disease: A study targeting substantia nigra in QSM imaging*

# Supplementary Figures and Tables

## Supplementary Tables

|  | Parkinson’s disease | Healthy controls |
| --- | --- | --- |
| Sex, man: woman | 41:46 | 24:29 |
| Age, year | 60.9±8.1 | 62.9±7.1 |
| Mini-Mental State Exam | ≥24 | ≥24 |

**Table 1.** Demographic and clinical characteristics of Parkinson’s disease and healthy control groups. Age values represent mean ± standard deviation.
